# Trends of rheumatoid arthritis monitorization in Romania

**Published:** 2010-08-25

**Authors:** C Mogoşan, V Stoica, C Mihai, C Ciofu, M Bojincă, M Milicescu, C Ciofu, V Crişan, M Banciu

**Affiliations:** * ‘Carol Davila’ University of Medicine and Pharmacy Bucharest; ‘Dr. Ion Cantacuzino’ Hospital – Department of Internal Medicine and Rheumatology Romania; ** Municipal Hospital Timisoara – Ambulatory of Rheumatology Romania; *** Rehabilitation Clinic – TimisoaraRomania

**Keywords:** rheumatoid arthritis, DMARDs non biologic, DMARDs biologic, monitorization, guideline

## Abstract

**Background**: rheumatoid arthritis (RA) is associated with the loss of overall functionality, which leads to substantial economic losses. Second line agents used in RA treatment require careful monitorization in terms of efficiency and tolerability.

**Objective**: trends, predictive factors and characteristics of clinical, biological and radiological RA monitorization in a cross sectional observational cohort study, conducted on over 206 patients in Romania, with a 12 months follow up (December 2007 – December 2008).

**Method**: Cases were recruited from the south–west region of the country, covering a geographical area of 23 counties. Patients were invited to complete three sets of interviews (collected by post) in a consent letter, containing self reported questionnaires, at 6 months intervals: an original questionnaire (which included quantitative self reported of pain, disease activity and fatigue on visual analogue scale – VAS), Health Assessment Questionnaire – HAQ – Disability and Discomfort Scales and EUROQOL EQ–5D, validated in Romanian (obtaining a user agreement by authors of the original version).

**Results**: analysis was carried out in SPSS 10. The cohort enrolled 206 patients, with the average age of 54.90 ± 12.67 years, 66% urban, 86.4% women, 29.1% professionally active, 48.5% graduates of primary education. The average disease duration after diagnosis of RA was of 9.40 ± 8.87 years. The duration of the treatment reported at baseline was of 2.70 ± 2.64 years. Most patients followed a program of monthly monitoring at a general practitioner (GP) (41.7% at baseline and 37.1% to 12 months). Visits to the rheumatologist followed a monthly regimen (32.3% at baseline and 31.7% to 12 months) or a 2 months interval (19.4% at baseline and 29.6% to 12 months, p = 0.000). Biological monitoring was quarterly (39.6% and 53.2% at 12 months; p = 0.000) or at 2 months interval (26.2% at baseline and 16.7% to 12 months, p = 0.000). X–ray monitoring lacked in over half the cases in a year of disease progression (63.3% at 6 months and 62.2% at 12 months), although it sums between 1 and 3 radiographs to one third of the cases (36.8%).

Conclusion: generally, in our country, there is a lack of aggregation in the dispensarization algorithm of patients with RA; consequently, the decision is awarded to the human factor. Under these circumstances, some patients are over evaluated. Promoting a dispensarization guide for RA patients could induce benefits both clinically and economically. Therefore, we submit a proposal of recommendations as a guideline for clinical, biological and radiological monitoring, according to the phase and stage of RA.

## Background

Rheumatoid arthritis (RA) is a chronic inflammatory systemic disease, with a fluctuating evolution and an unpredictable prognosis [4]; it causes severe decline of functionality and quality of life, increased morbidity and mortality [3].

The RA treatment uses non-biological and biological DMARDs (disease modifying anti-rheumatic drugs) with significant roles in reducing and preventing joint damage and improving the quality of life [1,6].Potential adverse events of these preparations require a rigorous monitoring. The aims seek to assess disease activity and therapeutic strategy adjustment, but also to prevent excess morbidity and hospitalizations related to tolerability [2]. 

Promoting an adequate monitoring program for RA patients is still a matter of international medical debate. [7]. 

For a definite RA, ACR recommendations (American College of Rheumatology) provide that medical visits to the primary care network are between 3 and 6 months, and to the rheumatologist between 6 and 12 months [5]. 

As presented in the end of the article regarding the Romanian RA population ‘X–ray’ PR, we return with aspects of the resource utilization, focusing on RA monitorization, during a period of 12 months follow up.

## Study Objective

The study aims to estimate the characteristics, trends and predictive factors of RA clinical, biological and radiological monitoring, in a cross–sectional, observational cohort study, conducted on over 206 patients in Romania, during 12 months follow up (December 2007 – December 2008). 

## Methods|materials

The cases (n = 206) came from two sources. Some cases are hospitalized patients in the ‘Dr. Ion Cantacuzino’ Hospital –Bucharest, Department of Internal Medicine and Rheumatology, during the year 2007. They have been drawn out chronologically, according to their time presentation, from the hospital electronic database. Inclusion criteria consisted of RA diagnosis and exclusion criteria and the presence of any malignancy. To cover a broader geographical area, other cases from the ambulatory care have been enrolled, by working with other rheumatologists in the country (randomly chosen from their patients lists, based on the same inclusion and exclusion criteria). 

RA diagnosis was established by the rheumatologist, for each patient apart. 

Through a consent–letter, we collected three series of interviews by mail, containing the patients' self–assessments, at 6 months intervals: T1 (at baseline), T2 (at 6 months) and T3 (at 12 months) – between December 2007 and December 2008. Because of those who failed the interviews no. 2 (T2) and no. 3 (T3), the initial size of the cohort was decreased, but statistically insignificant. These patients were reported as lost to follow up at T2 and T3. Thus, from 206 cases recruited initially (at T1), at T2 a number of 193 subjects responded, and, at T3, 189. 

Each set of assessments consisted of three different questionnaires: an original questionnaire, the Health Assessment Questionnaire (HAQ) Disability and Discomfort Scales and EUROQOL EQ–5D, validated in Romanian (with the consent for use from the authors of the original version). 

The collected data were distributed in variable categories: socio–demographic, comorbidities, general and functional disease features, quality of life and economic impact. 

## Data analyses

The sample (n = 206) covered 23 counties, from the southern and western areas of the country, which led us to estimate that the group is representative for the entire RA population in our country. 

The data have been analyzed in the program SPSS 10; we used ANOVA, two independent samples T test – for the continuous variables, Chi–Square, Kruskall Wallis and Man Whitney tests– for non–continuous variables, bivariate correlations (Pearson, Spearman coefficients), and linear regression.

The sample has been subdivided according to the therapy: one group was treated with non–biological DMARDs (monotherapy and combination = group A) and one group with biological DMARDs (group B). 

At T1, five patients were not on remission therapy, at T2, 4 patients, and, at T3, 11 cases. These subjects were excluded from the comparative analysis between groups. 

At the assessment times, the cohort groups consisted of at T1, group A = 129 cases, group B = 70 cases, at T2, group A = 120 cases, group B = 69 cases, at T3, group A = 108 cases, group B = 70 cases. 

## Results and discussions

The cohort baseline characteristics are listed in Table 1.

**Table 1:** Demographic characteristics at baseline (Results are given in average± DS for continuous variables and in percentages for non–continuous variables; a + b = 199 (7 cases have been excluded after splitting the sample into therapeutic groups); group A= non–biologic DMARDs; group B= biologic DMARDs; * Level of significance alpha: p<0,05; NS=non statistically significant )

Characteristic	Sample n = 206	Group A^a^ n = 129	Group B^b^ n = 70	p value Group A versus B
Age at inclusion (years)	54.90 ± 12.67	56.76 ± 12,25	51,84 ± 11,82	0,007*
Women	86,4%	88,4%	84,3%	NS
Urban	66%	64,3%	67,1%	NS
Married	75,7%	79,8%	71,4%	NS
Ethnicity (Romanian)	93,7%	93%	95,7%	NS
Professional activity				
Working active	29,1%	29,5%	30%	NS
Retired	69,4%	69%	70%	NS
Unemployed	1,5%	1,6%	–	NS
Education				
Not school education	1%	1,6%	–	NS
Primary education level	48,5%	49,6%	48,6%	NS
Medium education level	36,9%	37,2%	34,3%	NS
Superior education level	13,6%	11,6%	17,1%	NS
Monthly income				
< 500 lei	61%	60,5%	62,9%	NS
500 –1000 lei	29,3%	31%	25,7%	NS
1000 – 1500 lei	7,8%	7%	10%	NS
> 1500 lei	2%	1,6%	1,4%	NS
Disease duration starting diagnosis (ys)	9,40 ± 8,87	8,24 ± 8,93	11,32 ± 8,30	0,01*
Mean duration of current treatment (ys)	2,70 ± 2,64	2,71 ± 2,86	2,71 ± 2,19	NS

Indicators of resource utilization in the field of RA cases monitorization are given in Table 2.

**Table 2:** Indicators of clinical, biological and radiological monitoring (Results are given in average± DS for continuous variables and in percentages for non–continuous variables; a: non–biologic DMARDs; b: group B= biologic DMARDs; * Level of significance alpha: p<0,05; ** level of significance alpha < 0,01; T1 = baseline; T2= at 6 months; T3 = at 12 months; NS=non statistically significant )

Characteristic	T1 n = 206	T2 n = 193	T3 n = 189	p
Medical visits: rheumatologist/ 6 months	4,58 ± 2.67	3,74 ± 2,06	3,82 ± 2,17	0,000**
6 visits	32,3%	31%	31,7%	
3 visits	19,4%	31%	29,6%	<0,01**
2 visits	15,4%	14,4%	17,2%	
0 visits	3%	5,3%	4,8%	
Other	29,9%	18,3%	16,7%	< 0,01**
Medical visits: rheumatologist/ 12 months				NS
Group A^a^	11,45 ± 5,57	11,45 ± 5,57	11,45 ± 5,57	
Group B^b^	12,32 ± 5,86	12,32 ± 5,86	12,32 ± 5,86	
Medical visits: general practitioner/6 months	4,49 ± 2,84	3,89 ± 2,37	4,24 ± 2,14	<0,01**
6 visits	41,7%	39,5%	37,1%	
3 visits	7,5%	17,3%	24,7%	< 0,01*
2 visits	8,5%	9,7%	13,4%	
0 visits	15,6%	13%	3,8%	< 0,01*
Other	26,7%	20,5%	21%	
Medical visits: general practitioner/12 months				NS
Group A^a^	11,88 ± 5,85	11,88 ± 5,85	11,88 ± 5,85	
Group B^b^	12,60 ± 6,34	12,60 ± 6,34	12,60 ± 6,34	
Lab sets/ 6 months	2,60 ± 1,40	2,29 ± 1,23	2,23 ± 1,16	0,000**
3 sets	26,2%	19,1%	16,7%	
2 sets	39,6%	47,3%	53,2%	< 0,01**
1 set	16,8%	17,6%	19,4%	
0 sets	1%	3,7%	1,6%	
Other	16,4%	12,3%	9,1%	
Lab sets/ 12 months				0,08
Group A^a^	6,69 ± 2,99	6,69 ± 2,99	6,69 ± 2,99	
Group B^b^	7,50 ± 3,07	7,50 ± 3,07	7,50 ± 3,07	
X–rays number/ 6 months	1,45 ± 1,49	0,80 ± 1,24	0,72 ± 1,09	0,000**
>3 X–rays	9,4%	3,7%	1,1%	< 0,01**
1 – 3 X–rays	50,7%	33%	36,8%	< 0,01**
0 X–rays	39,9%	63,3%	62,2%	< 0,01**
X–rays number/ 12 months				< 0,01**
Group A^a^	3,29 ± 2,89	3,29 ± 2,89	3,29 ± 2,89	
Group B^b^	2,26 ± 2,74	2,26 ± 2,74	2,26 ± 2,74	

**Figure 1:**
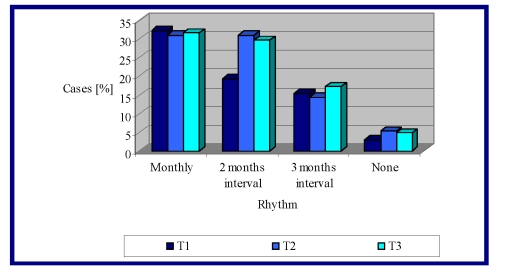
Rheumatologist monitorization during the interval T1 – T3

Concerning the rheumatologist' case monitoring, there is a fluctuation in the frequency of medical visits, within 12 months of follow up (Figure 1). 

Thus, more than one third of RA cases reported monthly, which represent the medical visits to the rheumatologist, were maintained at a constant level (32.3% – 31% – 31.7%). Medical checks with a regularity of 2 months were significantly increased in the interval T1 – T3 (19.4% – 31% – 29.6%, p <0.001). A slightly upward trajectory notes medical exams at every 3 months (15.4% – 14.4% – 17.2%).

The lack of compliance, assessed by the proportion of patients who did not submit to rheumatologists, increased slightly from T1 to T2 and T3, from 3% to 5.3% and 4.8% (Figure 1). 

In this context of lack of monitoring differentiation according to RA phase or stage, the most plausible hypothesis is that the rate of medical checks, involves the human factor. Furthermore, another approach brings to attention the fact that a closer follow up is responsible for a better efficiency in the evaluated parameters.

**Figure 2:**
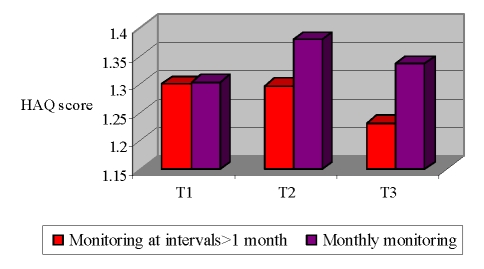
HAQ score evolution based on clinical monitorization rate

In this latter instance, we present Figure 2, which reproduces the comparative evolution of HAQ score in the interval T1 – T3, between cases monitored monthly and other cases that received a more spaced rate of monitorization.

Lack of improvement in HAQ score for patients receiving a more intensive clinical surveillance in terms of rate, confirms the human factor intervention in the decision on rhythm of clinical monitoring of RA patients.

**Figure 3:**
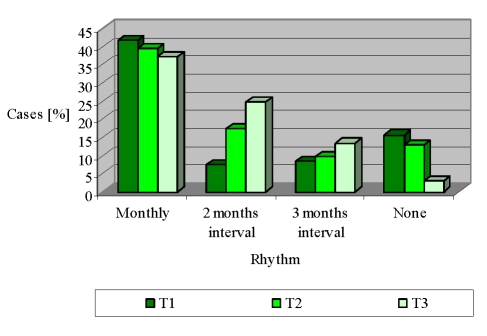
General practitioner' monitorization during the interval T1 – T3

Which is the contribution of the primary medical network in the monitorization of RA patients? The results dynamics is much wider. According to Figure 3, more than one third of the cases reported medical visits monthly, with a small reduction at T3 (41.7% – 39.5% – 37.1%). Medical checks with regularity of 2 months intervals have tripled in volume between T1 and T3 (7.5% – 17.3% – 24.7%, p <0.05). The same issue is seen ascending for medical checks at every 3 months (8.5% – 9.7% – 13.4%, p <0.05). 

Only at T3 is there an association between primary care monitoring rhythm and the self reported pain, disease activity and fatigue (on VAS) (r = 0.25, p = 0.000). However, there is a constant significant association between the visits to the primary care network and to the rheumatologist (ps = 0.345, p = 0.000). When the hypothesis that patients call on the general practitioner (GP) appears, the assessments on a monthly basis are the same receiving monthly rheumatologist evaluation.

A positive aspect is noted in patients who did not receive medical evaluation: their proportion has declined dramatically from 15.6% at T1 to 3.8% T at T3 (p <0.05) (Figure 3).

**Figure 4:**
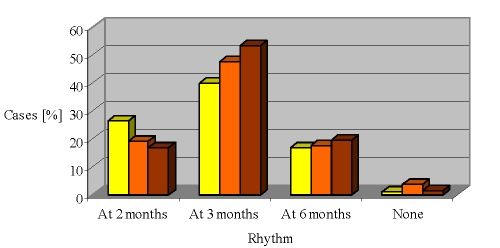
Biological monitoring dynamics

The dynamics of the biological monitoring describes a change in parameters, which suggests some trends (Figure 4).

After 12 months of follow–up, one tends to focus on biological exploration within 3 months (for 53% of cases), while the extremes range include a significant percentage of cases (16% to 20% at 2 months and 6 months). 

There is a constant direct correlation between the rate of biological monitoring and the medical visits to the primary network (ps = 0.228, p <0.01) and to the rheumatologist (ps = 0.345, p <0.001). Linear uni – and multivariate regression is attributed predictability only to the rheumatologist visits for the biological monitoring (ㆲ = 0.209, t = 2.8, p <0.01).

**Figure 5:**
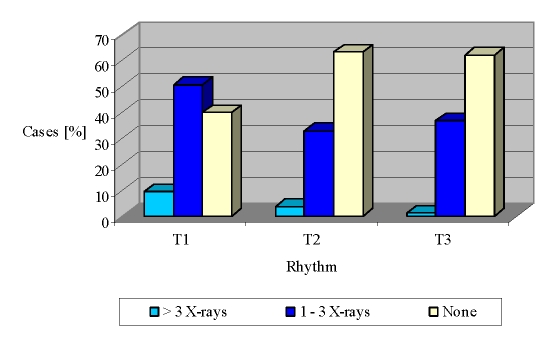
X–rays monitoring dynamics

The standardization of the osteoarticular lesions visible by standard radiography of hands and feet allow quantification of RA status and assess disease progression (using international standardized X–rays scores: Sharp – Van der Hejide score). In the study population at the end of observation (T1 – T3), over half the cases did not benefit from this imaging tool in the assessment of the disease lesions (T1: 39.9%; T2: 63.3%; T3: 62.2 %; p <0.001), while the remaining percentage of patients carried out between 1 and 3 radiographs (hands, feet and frequently lumbar spine) (Figure 5).

There are direct correlations between radiological and biological examinations at T2 and T3 (ps = 0.280, respectively ps = 0.228, p <0.01). There is a constant association of the radiological exploration with hospitalization (ps = 0.394, p <0.001), with visits to the rheumatologist, but not with visits to the primary re network (ps = 0.258, p <0.001).

**Figure 6:**
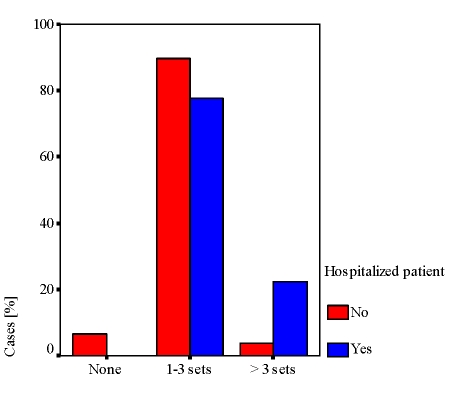
Biological monitoring based on hospitalization

**Figure 7:**
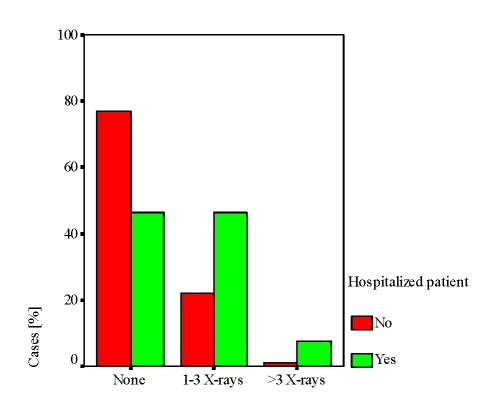
X–rays monitoring based on hospitalization

Generally, hospitalization is predictive for the biological evaluation (ㆲ = 0.4, t = 4.5, p <0.001) as for the radiological one (ㆲ = 0.35, t = 3.8, p <0.001). Cases monitoring mainly based on patients’ admission in the hospital is reproduced in Figure 6 and Figure 7. 

Biological exploration (over 3 sets) and radiological evaluation (1–3 X–rays and more than 3 X–rays) dominate in hospitalized patients compared with those who are not hospitalized. 

Looking towards the ambulatory system (outpatient care), there is a homogeneous relationship in both groups between visits to the rheumatologist and performing laboratory investigations (ps = 0.28, p <0.05) and X–ray evaluation (ps = 0.26, p <0.05). Only in group A, visits to the primary care network is correlated with laboratory tests (ps = 0.21, p <0.05). The lack of similar relationships in group B, suggests a closer monitoring of patients treated with biological agents directly by the rheumatologist (patient preference? / decision of the physician?), mostly through hospitalization services, compared with patients treated with non-biological DMARDs . 

In both groups, the disability level has no association or predictability role for the rhythm of primary care or rheumatologist monitoring. We say that variables have an independent relation between each other. 

Trying to determine which are the causes that induce the monitoring rhythm in controls to GP or rheumatologist, we analyzed the predictability of patients' self–evaluations VAS (pain, activity fatigue) and the addressability to primary medical care and rheumatologist. Uni–and multivariate linear regression show no predictive indicators for calling the healthcare services. 

Having noted these data, we can say that an unequivocal role in setting the RA monitoring rate rely on the human factor. In addition, there is evidence that the medical examination laboratory tests and possibly X–rays are recommended together: the rheumatologists' visits are associated with performing laboratory tests and X–ray evaluation, in both groups (ps = 0.33, p <0.01), respectively (ps = 0.35; p <0.01). These relations also have a predictability potential (for biological exploration: t = 5, ㆲ = 0.34, p <0.001; for X–ray evaluation: t = 3.6, ㆲ = 0.26, p <0.001). 

## Conclusions

### Clinical monitoring of RA patients to rheumatologists has no correlations with disease–related parameters

The visit rate has increased in volume, although the evaluated disease parameters have not worsened (HAQ score, quantitative self reported on VAS, utility, EQ–5D VAS). In the same direction, we mention no association between visits to rheumatologist and: HAQ category, hospitalization, self–reported pain, fatigue and disease activity (on VAS), increased consumption of NSAIDs or corticosteroids medication. In the absence of differentiating clinical monitoring according to the RA stage or phase, the most plausible hypothesis involves the human factor in determining the tempo of medical checks. The lack of improvement in HAQ scores for patients who received more intensive clinical surveillance (in terms of its rhythm), confirms the decision of the physician on clinical monitoring of RA patients. 

### Clinical monitoring of RA patients by GPs has a monthly trend, without distinction based on disease features.

Except for the direct correlation observed only at T3, between primary care network monitoring and self reported pain, disease activity and fatigue (on VAS), any other disease parameters were not associated with GP visits. As a result, patients do not seem to appeal for medical advice following an unfavorable disease evolution, or the proportion of those who fall into this category is extremely small. Interestingly, it is noted that there is a constant significant association between visits to the primary care network and rheumatologist (ρs = 0.345, p = 0.000). It appears that the hypothesis that RA patients who require monthly GP evaluation are similar to the ones receiving monthly rheumatologist evaluation. Because predictive factors belonging to RA or related disorders have not been observed, it appears that for the entire cohort, the decision regarding the rate of medical monitoring belongs in a large proportion to the clinician (GP or rheumatologist). 

### Biological monitoring of RA cases is not differentiated by disease factors

There is a constant direct correlation between the rate of biological monitoring and medical visits in both the primary care network (ps = 0.228, p <0.01) and Rheumatology (ps = 0.345, p <0.001). The evaluation of both values in relation to their predictive ability for biological monitoring rate, shows that only visits to rheumatologists are predictable for biological evaluation (ㆲ = 0.209, t = 2.8; p <0.01). In other words, patients who are presented in a certain pattern in medical practices are oriented to the biological exploration according to this rate. This kind of relationship is predictable, but has a strong particularity because of the lacking associations between rate of biological exploration and disease factors. Again, the clinician's decision appears in the foreground. 

### Radiological monitoring of cases is insufficient.

At the end of the observation period, more than half the cases did not benefit from an X–ray examination to directly assess disease impact (62.2%). We consider that an unfavorable aspect in the disease monitoring. 

There is a significantly divergent behavior according to the therapeutic groups: the patients receiving biological agents are more closely biologically monitored (annual average of biological controls 7.50 vs. 6.69 in group A, p = 0.08); on the other hand, the patients treated with non biological DMARDs are more intensively radiological monitored (3.29 annual X–rays compared with 2.26 in group B, p = 0.01). 

We remind that the RA population is over 9 years of disease duration and more than 2 years of stable background therapy, exceeding early stage of disease, which would require a more intensive surveillance

All this generally shows an emphasis on clinical and biological monitoring parameters, amid a lack of aggregation in monitoring algorithm of RA patients in our country. 

Biological evaluation allows the assessment of tolerability (blood, liver and kidney) and provides relations of efficiency (dynamic of the inflammatory syndrome). These data, however, occupy a second position in the quantification of the therapeutic effectiveness, the clinical evaluation (absolutely necessary) being in the foreground. An extensive biological monitorization (at the expense of clinical and radiological) would not conduct to a therapeutic benefit.

**Figure 8:**
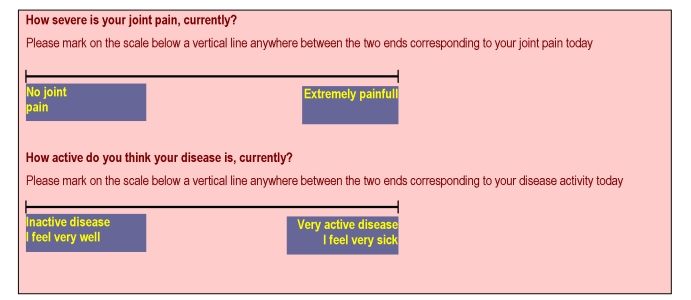
Self reported pain and disease activity through VAS

In order to increase the effective monitoring of RA patients and to diminish the influence of human factor, we suggest the following guidelines as recommendations:

- **Rate of the RA biological assessment**: given the risk of drug toxicity at the start of RA therapy, during the first three months of treatment, we suggest a monthly biological monitoring. Subsequently, after this period, the biological monitoring should be done at intervals of 3 months and whenever required by the clinical evolution. In this framework, the minimum biological package relating to efficiency and tolerability include: CRP or ESR, complete blood count, ALT, creatinine, urinalysis;
- **Rate of the RA radiological assessment**: for non–erosive disease, plain X–rays of hands and feet at 6 months intervals; during erosive RA, plain X–rays of hands and feet at intervals of 1 year. It is recommended that X–ray lesions are quantified by using a standardized international radiological RA score (Sharp, Van der Heijde).
- **Rate of RA clinical evaluation**:
  - It is recommended that the rate of clinical monitoring in the primary care network (GPs) is overlapped to the biological rate: the first 3 months of starting RA therapy – monthly and quarterly thereafter. If any adverse events related to tolerability occur, the patient will be referred to the rheumatologist;
  - In the network of specialized rheumatology: during any active RA the clinical evaluation should be done at 3 months intervals plus any other time, as clinically required; during inactive disease, biannual clinical assessments. It is recommended that the clinical evaluation includes both quantitative self reported of pain and disease activity, through VAS (0 to 100 mm, marked the extreme) (Figure 8).
